# Supplementary figures and images for: Molecular systematics of the subfamily Limenitidinae (Lepidoptera: Nymphalidae)
Source: PeerJ. 2018 Feb 2;6:e4311. doi: 10.7717/peerj.4311 (PMC5798401; doi:10.7717/peerj.4311)

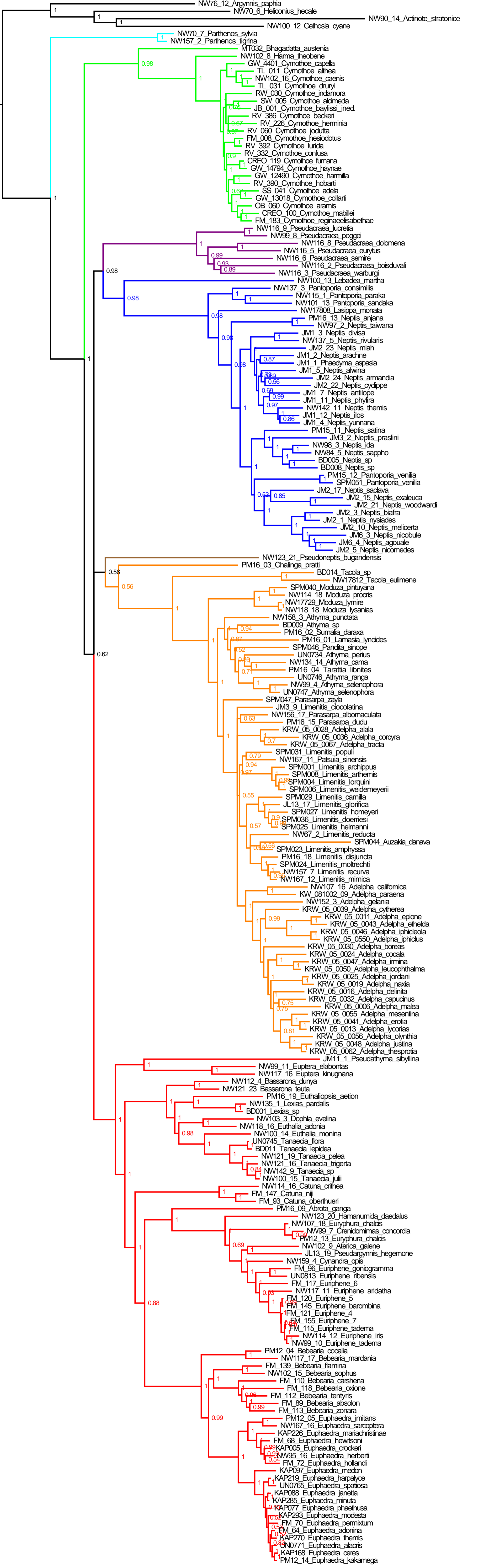

0.03

Supplement: Fig. S1 — Major lineages that are considered tribes in this paper are coloured as in Fig. 1. [file peerj-06-4311-s002.pdf]

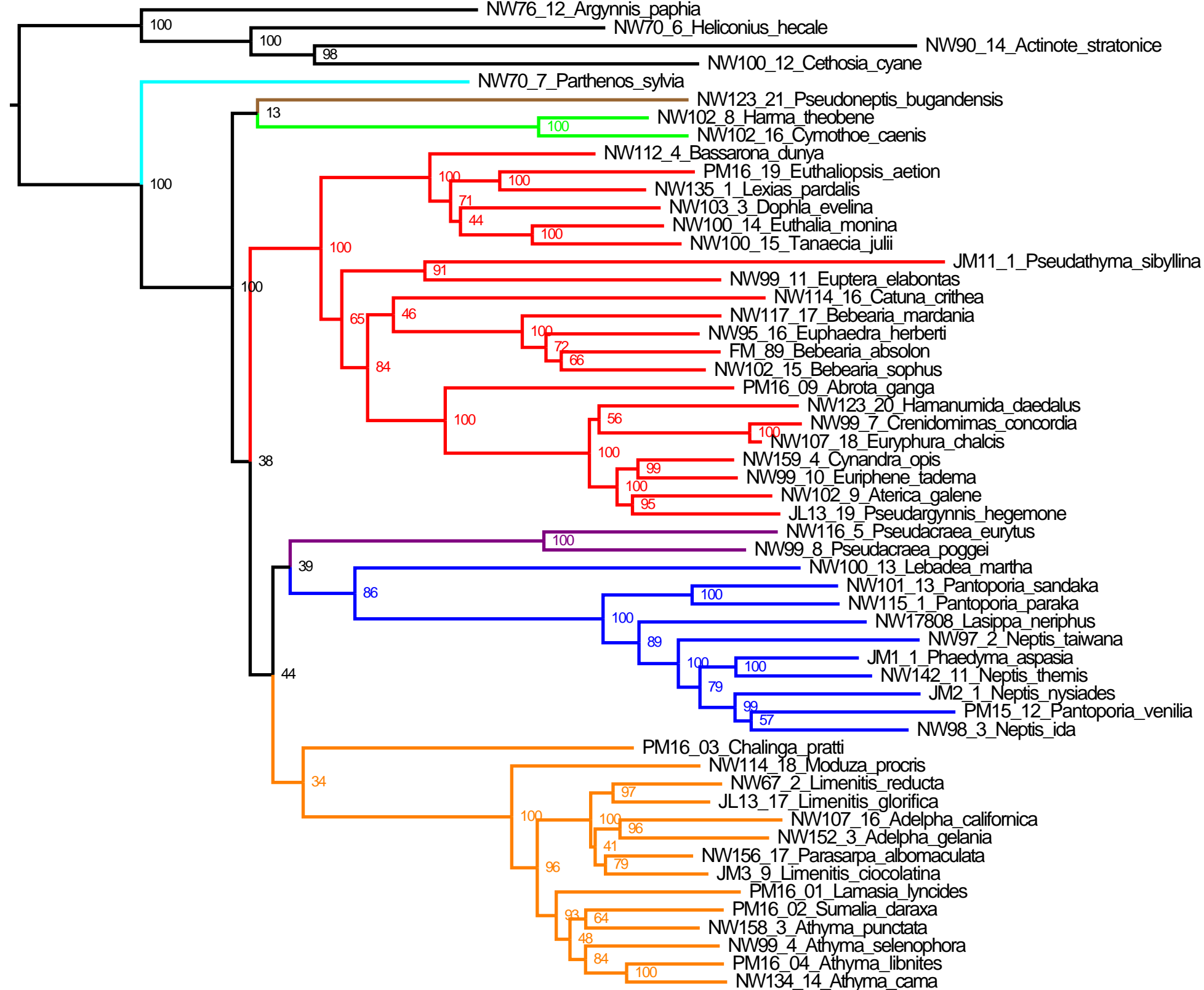

0.03

Supplement: Fig. S2 — Values to the right of nodes are bootstrap values for that node. Major lineages that are considered tribes in this paper are coloured as in Fig. 1. [file peerj-06-4311-s003.pdf]
